# Androgen Receptor Is Dispensable for X-Zone Regression in the Female Adrenal but Regulates Post-Partum Corticosterone Levels and Protects Cortex Integrity

**DOI:** 10.3389/fendo.2020.599869

**Published:** 2021-01-21

**Authors:** Anne-Louise Gannon, Laura O’Hara, Ian J. Mason, Anne Jørgensen, Hanne Frederiksen, Michael Curley, Laura Milne, Sarah Smith, Rod T. Mitchell, Lee B. Smith

**Affiliations:** ^1^MRC Centre for Reproductive Health, University of Edinburgh, The Queen’s Medical Research Institute, Edinburgh, United Kingdom; ^2^School of Environmental and Life Sciences, Faculty of Science, University of Newcastle, Callaghan, NSW, Australia; ^3^Centre for Discovery Brain Sciences, Hugh Robson Building, George Square, Edinburgh, United Kingdom; ^4^Department of Growth and Reproduction, Rigshospitalet, University of Copenhagen, Copenhagen, Denmark; ^5^International Centre for Research and Research Training in Endocrine Disruption of Male Reproduction and Child Health (EDMaRC), Rigshospitalet, Copenhagen, Denmark

**Keywords:** adrenal cortex, X-zone, androgen receptor, spindle cell, hyperplasia

## Abstract

Adrenal androgens are fundamental mediators of ovarian folliculogenesis, embryonic implantation, and breast development. Although adrenal androgen function in target tissues are well characterized, there is little research covering the role of androgen-signaling within the adrenal itself. Adrenal glands express AR which is essential for the regression of the X-zone in male mice. Female mice also undergo X-zone regression during their first pregnancy, however whether this is also controlled by AR signaling is unknown. To understand the role of the androgen receptor (AR) in the female adrenal, we utilized a *Cyp11a1*-Cre to specifically ablate AR from the mouse adrenal cortex. Results show that AR-signaling is dispensable for adrenal gland development in females, and for X-zone regression during pregnancy, but is required to suppress elevation of corticosterone levels post-partum. Additionally, following disruption to adrenal AR, aberrant spindle cell development is observed in young adult females. These results demonstrate sexually dimorphic regulation of the adrenal X-zone by AR and point to dysfunctional adrenal androgen signaling as a possible mechanism in the early development of adrenal spindle cell hyperplasia.

## Introduction

The adrenal cortex has a complex multi-cellular structure comprised of specialized concentric zones that support the biosynthesis of steroids. The zones that make up the adrenal cortex consist of the zona glomerulosa (ZG), zona fasciculata (ZF), and the zona reticularis (ZR). These are responsible for the production of aldosterone, cortisol and androgens, respectively, which act both locally and systemically to support development and physiological function (1, 2). Adrenal androgens account for approximately 25% of circulating androgens in women (3), with cross-sectional analyses revealing that decreased circulating androgens impact health and wellbeing in both young and ageing women (4, 5). Outside of the adrenal gland, adrenal androgens are fundamental mediators of ovarian folliculogenesis (6), embryonic implantation (7), and breast development (8), however, the role androgen-signaling plays within the adrenal gland remains unclear. The adrenal cortex expresses the androgen receptor (AR) (9, 10) making the adrenal a target of androgen signaling as well as a site of androgen production.

The mouse has largely been overlooked as a model organism for studying the complexities of androgen-signaling in adrenal development and function, possibly because the mouse adrenal lacks CYP17A1 expression and thus unlike the human adrenal, does not, itself, produce androgens. However, regardless of species differences in the source of androgens, there is strong evidence that, as in humans, the mouse adrenal gland responds to androgen signaling. In both the male and female mouse, the adrenal develops a transient cortex zone termed “X-zone”, thought to be homologous to the fetal zone of the human adrenal. During postnatal development, X-zone involution occurs during puberty in males and at first pregnancy or old age in females who have not had a pregnancy (nulligravida) (11, 12). Studies conducted in female mice show that the X-zone regresses following treatment with testosterone-propionate (13), suggesting that androgens, although not produced by the mouse adrenal, can regulate the female adrenal cortex. Building on this work, we have recently demonstrated the role of androgen-signaling *via* AR in the male adrenal, showing that AR is essential for X-zone regression during puberty and to protect the adrenal against age-related degeneration and spindle cell hyperplasia (14). However, the specific roles of androgen signaling *via* AR in female adrenals remains unknown.

In this study, we have used a novel knockout model targeting the AR in the adrenal gland of female mice to establish whether androgen-signaling *via* AR in the adrenal cortex, controls cortex remodeling and X-zone regression in females similar to our observations in males. This study identifies AR as an essential regulator of adrenal cortex maintenance provides a significant novel entry point into advancing our understanding of X-zone regulation and sex-biased adrenal disease in women.

## Materials and Methods

### Ethics Statement

All mice used in experiments were under a strict standard of care and experimental planning covered by licensed approval from the UK Home Office (License number 70/8804) held by L.B.S.

### Targeted Ablation of Androgen Receptor From the Adrenal Cortex Using *Cyp11a1*-GC Cre

To specifically ablate AR from the female adrenal cortex, Cre/*loxP* technology was used. Male C57BL/6 mice carrying a random insertion of the *Cyp11a1*-GC Cre (15) were mated to C57BL/6 female mice homozygous for floxed AR (16). Male offspring were either *Cyp11a1*^+/GC^; AR^fl/y^ mice with adrenal androgen receptor ablation termed “G1 Ad-ARKO” or *Cyp11a1*^+/+^; AR^fl/y^ “control” littermates. Females from this first generation breeding were mosaic for AR in the adrenal due to X-linkage. To fully ablate AR from the female adrenal cortex G1 Ad-ARKO males were mated to C57BL/6 female mice homozygous for floxed AR. Offspring from these breeding were termed “G2 Ad-ARKO” or “control” littermates depending on their inheritance of the Cre transgene. Mice were culled and tissue collected at day (d) 80.

### PCR Genotyping of Mice

Genotyping for the inheritance of Cre recombinase was performed as previously described (17). PCR amplification products were resolved using the QIAxcel capillary system (QIAGEN, Crawley, United Kingdom). An amplicon of 102bp indicated the inheritance of the Cre recombinase transgene.

### Collection of Post-Partum Females

G2 Ad-ARKO females were set up in mating pairs with wild-type (WT) C57BL/6 males. Females were allowed give birth and were culled 24 h post birth. A control group of WT pregnant females were collected under the same conditions. Females gave birth between the ages of d80 and d90.

### Tissue Collection and Processing

Mice were culled by inhalation of carbon dioxide and death was confirmed *via* cervical dislocation. Body weight was measured, and adrenals were removed and weighed. Reproductive organs were also examined, removed and weighed. Tissues were fixed in Bouin’s fixative (Clin-Tech, Guildford, UK) for 4 h. Bouin’s-fixed tissues were processed and embedded in paraffin wax, and 5 µm sections were used for histological analysis. Sections of adrenals were stained with hematoxylin and eosin using standard protocols and examined for histological abnormalities.

### Immunohistochemistry

Immunolocalisation was carried out under various protocols. Single antibody colorimetric (DAB) immunostaining method, as described previously (18), a single or double antibody tyramide fluorescent immunostaining method, as described previously (15, 19), or Bond machine automated immunostaining method (Leica), as described previously (18). An exception to the described protocols is the H_2_O_2_ concentration, whereby washes were performed at 3% H_2_0_2_ TBS. Antibodies used are listed in **Table 1**. A minimum of five individual sections for each age and genotype were immunostained in each experiment.

**Table 1:** Immunohistochemistry performed in this study, listing antibody source and method used.

Protein stained for	Method	Antibody dilution	Primary antibodies
HSD3B	Single fluorescence	1:750	HSD3B: Santa Cruz #sc30820
AR	Single fluorescence	1:500	AR: Spring Bioscience #M4070
PCNA	DAB	1:200	Sigma #P-8825
AKR1B7	Single fluorescence	1:50	Santa Cruz Biotechnology #sc-27763
HSD20alpha	Single fluorescence	1:250	Aviva Systems Biology #OAGA00409
GR	DAB	1:200	Cell Signaling #12041
CASP3	Bond	1:100	CASP3: Abcam #ab4051

### Blood Collection and Serum Extraction

Immediately after culling, blood was collected from control and Ad-ARKO mice *via* cardiac puncture with a syringe and wide beveled needle to prevent lysis. Blood was collected in EDTA coated tubes to prevent coagulation and was spun down within 20 min of extraction. Plasma was separated by centrifugation at 13,000 revolutions per minute (rpm) for 10 min and stored at -80°C long-term.

### Quantification of Hormone Levels

Post-partum corticosterone was measured using a mouse corticosterone ELISA kit (Arbor Assays, KO14-H5) according to the manufacturer’s instructions. All samples were run as a single assay for each hormone. Females were collected at various stages throughout estrus and were not estrus synchronized.

Serum analysis of circulating androgens was achieved *via* new and sensitive isotope-dilution TurboFlow-LC-MS/MS method. Using this method we are able to quantify androstenedione, testosterone, progesterone, and corticosterone in human and mouse serum as previously described (20), without modifications. However, since the steroid concentration in several of the mouse samples were higher than the normal analytical range for steroids in human serum, the linearity of calibration curves was investigated by preparing calibration materials in synthetic serum expanded in the high concentration area by including a total of 23 different dilutions of the standard stock solution. The linear range of calibration curves for regression analysis are shown in **Table 2**. Control materials were based on two pools of mouse serum (**Table 2**) and different pools of human serum from children and adults, spiked in respectively low and high levels and not spiked serum pool. Samples for this study were analyzed in a series of seven batches, where each batch included standards for calibration curves, 60 blind samples, one blank, three un-spiked serum pool samples, three pool controls spiked at respectively low and high levels. The inter-day variation, expressed as the relative standard deviation (RSD) was ≤ 8% for all analytes in both spike levels. The recovery was > 89% for all analytes. **Table 2** shows the limit of quantification (20), the linear range for each individual calibration curve, and the inter-day validation. All LC-MS/MS analyses for the samples detailed in this study were performed at the Dept. of Growth and Reproduction, Rigshospitalet, Copenhagen University Hospital.

**Table 2:** LC-MS/MS limits of quantification (LOQ).

	Serum #72 Q Low			Serum #72 Q High			Serum #73 Q Low			Serum #73 Q High			LOQ	Range
	Mean (nM)	RSD (%)	Recovery (%)	Mean (nM)	RSD (%)	Recovery (%)	Mean (nM)	RSD (%)	Recovery (%)	Mean (nM)	RSD (%)	Recovery (%)	(nm)	(nM)
Estrone 1-sulfate	0.43	7	105	0.94	5.7	99	0.39	8.7	96	0.92	8.9	96	0.026	LOQ-10
Cortisone	4.63	0.87	100	10.6	7.6	98	4.2	4.1	91	10.4	3.4	97	0.19	LOQ-112
Cortisol	35.3	8.1	102	81	2.3	100	32.3	0.9	93	77.7	1.7	96	1.9	LOQ-794
Dehydroepiandrosterone sulfate	720	7.5	104	1652	2.7	102	671	1.1	97	1584	1.4	98	19	LOQ-3000
Corticosterone	38	7.3	107	46.5	12	109	18.3	8.7	103	25.5	0.54	98	0.1	LOQ-144
11-Deoxycotisol	1.86	2.3	104	4.07	6.8	98	1.78	1.9	96	4.02	3.4	95	0.017	LOQ-40
Δ4-androstenedione	2.13	0.74	102	4.72	4.2	97	3.39	6.8	94	5.98	3	93	0.042	LOQ-1746
Testosterone	5.62	1.1	111	7.71	3.1	99	49.3	3	106	51.4	3	98	0.012	LOQ-1732
17α-hydroxyprogesterone	2.09	11	96	4.53	7	95	2.18	6.3	90	4.67	0.36	93	0.1	LOQ-1513
Progesterone	3.48	1.3	100	7.99	4.9	98	3.89	5.5	92	8.33	5.2	94	0.036	LOQ-500

LC-MS/MS limits of quantification (LOQ) and range of calibration curves were based on 10 standards prepared as for human serum analysis (20). (B) LC-MS/MS validation: Inter day control materials for this study were prepared in two pools of mouse serum (serum #72 and #73) spiked in low (Q Low) and high (Q High) levels. Results are the mean (n=3) of control materials analyzed in three batches.

### Quantification of Spindle Cell Occurrence

Analysis of spindle cell occurrence was achieved *via* serial sectioning five adrenals per experimental group. Multiple sections were selected evenly throughout each adrenal, and H&E stained to carry out morphology analysis. Spindle cells are characterized based on their shape. Under a microscope, these cells look long and slender, distinguishing them from other cells in the cortex, which are round. When the presence of spindle cells was observed it was recorded, and the adrenal scored positive. The number of adrenals that were scored as positive was expressed as a percentage. For example, in five samples analyzed if only one sample presented with spindle cells it would equate to 20% occurrence for that genotype. These measurements accounted for presence/absence, not severity.

### PCNA and CLEAVED CASPASE 3 Positive Cell Counts

Analysis of proliferation and apoptosis was achieved *via* counts for PCNA and Cleaved Caspase 3 respectively. Each cohort had an n=5 and five sections from each animal was counted. Each section was scanned on the Axioscan to provide a whole section view. These sections were then counted for every PCNA/CLEAVED CASPASE 3 positive cell observed in that section in Zen lite (ZEISS).

### Statistical Analysis

Groups consisted of 5 mice for morphological and presence/absence analysis. Power calculations were carried out with the software Graphpad Statmate. Power calculations based on previous stereology work determined that a sample size of 5 is appropriate for quantitative end points for cell counting and immunohistochemistry. Statistical analysis is performed *via* GraphPad Prism (version 7; GraphPad Software Inc., San Diego, CA, USA). Statistical tests include a two-tailed unpaired t-test (if comparing two groups), a one-way ANOVA with Tukey’s post-hoc test (if comparing multiple groups), a two-way ANOVA with Tukey’s post-hoc test (if comparing multiple groups and variables), and Chi Squared test for determining proportion of a population Values are expressed as mean ± S.E.M.

## Results

### Confirmation of Ablation of Androgen Receptor From the Female Adrenal Cortex

**Figure 1:**
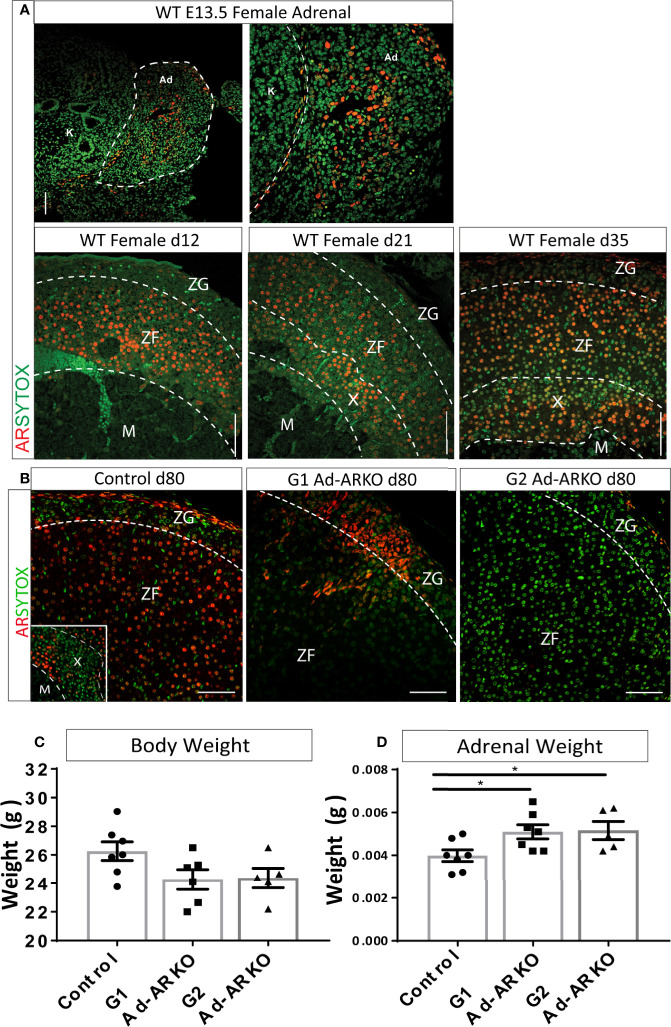
Confirmation of AR ablation in the adrenal cortex. **(A)** AR localization can be detected at e13.5 in the WT female adrenal, with staining localized to the central region of the fetal adrenal (N = 2). Immunostaining of AR during postnatal development shows localization from the inner most cortex/medulla boundary and ZF at d12 and d21. At d35, AR is observed in the X-zone, ZF and ZG (N = 3) **(B)** Immunostaining of AR in the adrenal cortex reveals partial ablation in the G1Ad-ARKO and complete adrenal AR ablation in the G2 Ad-ARKO females. Insert demonstrates AR localization in the X-zone of WT d80 Controls. Red: AR protein, Green: sytox counterstain. **(C)** Bodyweight analysis of d80 WT controls, G1Ad-ARKO and G2 Ad-ARKO females revealed no changes. **(D)** Adrenal weight analysis shows a significant increase in G1 and G2 Ad-ARKO mice compared to respective controls (one-way ANOVA; n=5-8, *p < 0.05, *p < 0.05, Tukey’s post-hoc analysis, error bars SEM). Annotations; X, X-zone; ZF, zona fasciculata; ZG, zona glomerulosa. Scale Bars 100 µm.

We have previously shown AR to be expressed in the adrenal cortex of human and male mice (14). To establish AR location within the female adrenal cortex, adrenals from wild-type (WT) females were examined in early embryonic development (e13.5) and early postnatal development (d12, 21, and 35). Analysis of AR localization in early embryonic development shows AR expression throughout the fetal adrenal. AR protein is also present at all postnatal time points examined. At d12 and 21, AR is present in the Zona Fasciculata (ZF) and X-zone. AR protein is present in all cortex zones by d35 (**Figure 1A**).

To determine whether adrenal cortex AR-deficient mice could provide a suitable platform to investigate the role of AR in the female adrenal cortex, we utilized a *Cyp11a1*-GC Cre line (15) to generate a mouse model with adrenal-specific AR ablation. We have previously demonstrated that this Cre line targets differentiated steroidogenic cells of the adrenal cortex while not targeting the adrenal capsule (15). Utilizing this model, we previously demonstrated ablation of AR from the male adrenal cortex (14). AR is located on the X-chromosome; therefore the first generation adrenal AR knockout (G1 Ad-ARKO) females are mosaic for presence/absence of AR expression in the adrenal cortex (due to random X-chromosome inactivation), while the G2 animals (2^nd^ Generation Ad-ARKO) completely lack AR from adrenocortical steroidogenic cells. Both generations of Ad-ARKO females are used in this study as mosaic ablation in G1 enables investigation of autocrine AR action inside cells within the adrenal cortex, in addition to establishing the effects of complete loss of adrenal AR *via* analysis of G2. Immunolocalization demonstrates AR is expressed in all zones of the adrenal cortex in controls, in contrast, mosaic ablation of AR from adrenocortical steroidogenic cells is observed in G1 Ad-ARKOs, with sporadic AR localization throughout the cortex and complete ablation from adrenocortical steroidogenic cells is observed in G2 Ad-ARKOs (**Figure 1B**). Following AR ablation, body weight was unchanged in any cohort compared to controls (**Figure 1C**). However, we observe an increase in adrenal weight in both G1 and G2 Ad-ARKO females compared to controls (**Figure 1D**). These data confirm the ablation of AR from the adrenal cortex, and that our model permits tissue-specific interrogation of AR signaling in the female mouse adrenal cortex.

### Ablation of Androgen Receptor Results in Disruption to Cortical Markers 20 Alpha-HSD and AKR1B7

**Figure 2:**
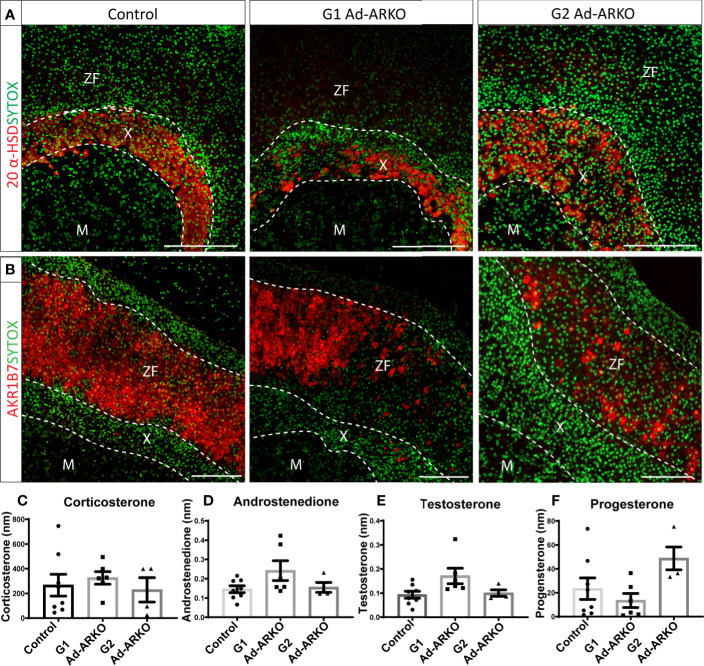
AR ablation results in disruption to adrenocortical markers. **(A)** Immunostaining of 20 α-HSD revealed an enlarged X-zone in G2 Ad-ARKO females up through the cortex, compared to WT controls were the X-zone is maintained at the cortex medulla boundary. Red: 20alpha-HSD protein, Green: sytox counterstain. **(B)** N=5. Immunostaining of AKR1B7 revealed loss of positive cells throughout the adrenal cortex in 1st and G2 Ad-ARKO females. Red: AKR1B7 protein, Green: sytox counterstain. N=5. Analysis of corticosterone **(C)**, androstenedione **(D)**, testosterone **(E)** and progesterone **(F)** reveals no changes following ablation of AR in the adrenal cortex (one-way ANOVA; n=4-8, Tukey’s post-hoc analysis, error bars SEM). Serum #72 and serum #73 were pooled serum from mouse spiked in two levels with androgens. All mice collected and analyzed at d80. Scale Bars 100µm. Annotations; M, medulla; X, X-zone; ZF, zona fasciculata.

Maintaining adrenal cortex zonation is essential for normal steroid production (21). An increase in adrenal weight in both generations of Ad-ARKOs, suggests expansion of, or perturbed, adrenal zonation. To investigate this, we examined key adrenal cortical markers including 20-α-hydroxysteroid dehydrogenase (MGI symbol AKR1C18, commonly termed, 20α-HSD, human homologue AKR1C1), a well-described X-zone marker (22), aldo-keto reductase family 1, member 7 (MGI symbol AKR1B7), a well-defined ZF marker known for detoxifying cholesterol cleavage products (23), and 3β-hydroxysteroid dehydrogenase (MGI symbol HSD3B, commonly termed 3β-HSD), a steroidogenesis marker expressed in both ZG and ZF (19). Analysis of 20α-HSD immunolocalization reveals that 20α-HSD positive X-zone cells are no longer restricted in a tight band in the usual X-zone location at the cortex-medulla boundary (the region where the adrenal medulla meets the adrenal cortex) (**Figure 2A**). Immunostaining shows disruption of AKR1B7 expression in both G1 and G2 Ad-ARKO females, although AKR1B7 can still be observed, large portions of the ZF no longer positive for AKR1B7 (**Figure 2B**). Furthermore, although disrupted, immunolocalization for both 20α-HSD and AKR1B7 demonstrate that a definitive adrenal cortex develops in G1 and G2 Ad-ARKOs. Despite this disruption to cortical markers, we see no changes to concentration of circulating steroid hormones; corticosterone, androstenedione, testosterone or progesterone (**Figures 2C–F**).

### Androgen Receptor Is Dispensable for X-Zone Regression During Pregnancy in the Female Adrenal

**Figure 3:**
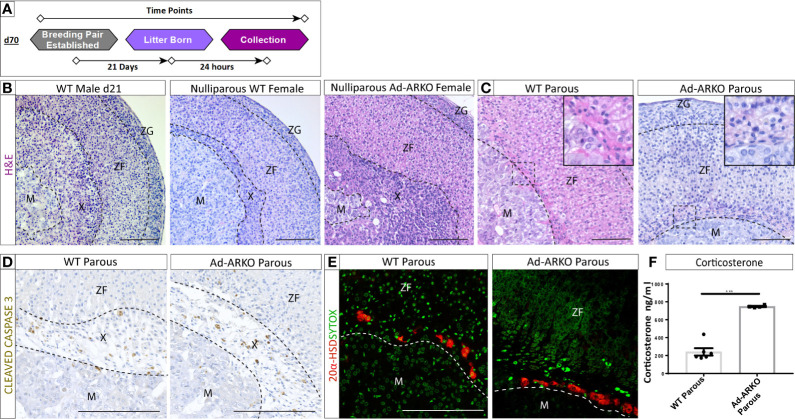
AR is dispensable for X-zone regression during pregnancy. **(A)** Diagram depicting collections of G2 Ad-ARKO females and controls following pregnancy for X-zone regression analysis. Nulliparous G2 Ad-ARKO females and Nulliparous WT controls were placed in a breeding pair at d70. Females were collected 24 h post-partum. **(B)** X-zones are present in WT d21 males and WT virgin females. N=5. **(C)** Analysis of X-zone morphology following pregnancy in WT and G2 Ad-ARKO females reveals regression of the X-zone in both cohorts. N=5. **(D)** Immunostaining of cleaved caspase 3 shows apoptosis of the X-zone in both WT and G2 Ad-ARKO females. N=5. **(E)** Immunostaining of 20 α-HSD localization shows only a few X-zone cells remaining in the cortex of WT controls and G2 Ad-ARKO females. Red: 20alpha-HSD protein, Green: sytox counterstain. N=5. **(F)** Analysis of circulating corticosterone shows a significant increase in G2 Ad-ARKO females following pregnancy (Students t-test; n=4–6), ***p < 0.05, error bars SEM) compared to WT controls. Mice collected between d80-90. Scale Bars 100 µm. Annotations; M, medulla; X, X-zone; ZF, zona fasciculata; ZG, glomerulosa.

The X-zone, which is typically confined to a tight band around the cortex-medulla boundary, was noted to occupy a large portion of the cortex (**Figure 2A**). Under normal conditions, the X-zone shows sexually dimorphic regression. In males, the X-zone is retained until d28 and then begins to regress (14). We recently demonstrated that this regression is an AR-dependent process (14). To determine whether androgen-signaling *via* AR also explains the normal X-zone regression observed during pregnancy (**Figures 3A–C**) (12, 24) we analyzed G2 Ad-ARKO females and littermate controls 24 h post-partum. G1 Ad-ARKO females were not used in this analysis as they still express AR. Surprisingly, analysis of morphology and X-zone markers reveal X-zone regression in both gravida (confirmed pregnancy) controls and gravida G2 Ad-ARKOs. (**Figures 3B–E**). X-zone regression involves apoptosis, and immunostaining of CLEAVED CASPASE 3 reveals apoptosis of the X-zone in both gravida controls and gravida G2 Ad-ARKOs (**Figure 3D**). Immunostaining for 20 alpha-HSD confirms removal of X-zone cells with only few positive cells remaining in both gravida controls and G2 Ad-ARKOs (**Figure 3E**).

A primary output for the adrenal cortex is the stress hormone corticosterone. Pregnancy results in a fluctuation of cortisol (humans)/corticosterone (rodents) that are integral in healthy pregnancy for the mother and offspring (25). Despite no changes in X-zone regression between controls and Ad-ARKOs during pregnancy, circulating corticosterone is significantly elevated in gravida G2 Ad-ARKO females compared to gravida controls (**Figure 3F**).

### Partial and Complete Ablation of Adrenal AR Results in Early Onset Spindle Cell Development in the Outer Cortex

**Figure 4:**
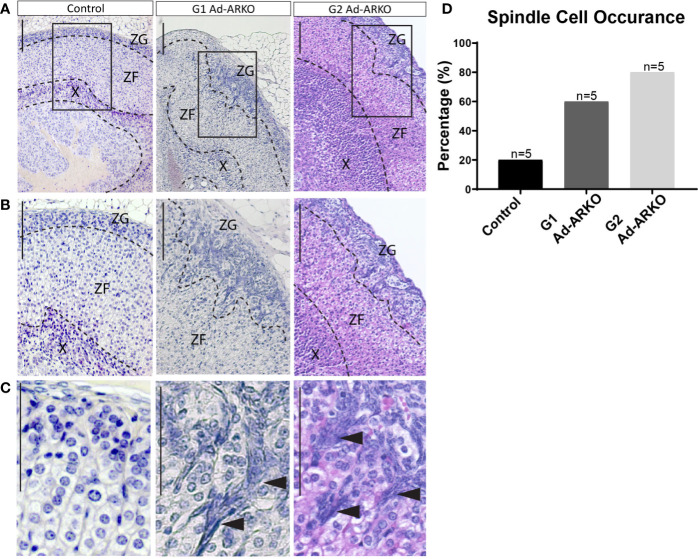
Spindle cell hyperplasia can be seen in G1 and G2 Ad-ARKOs. **(A–C)** H&E analysis reveals spindle cell lesions in the outer adrenal cortex that protrude down from the ZG into the ZF in G1Ad-ARKO females. These spindle cells can also be observed in G2 Ad-ARKO females but are more progressive and occupy a larger portion of the adrenal cortex. Spindle cells denoted by arrow heads **(C)**. N=5. **(D)** Analysis of spindle cell occurrence in Ad-ARKO females reveals a 60% occurrence in G1 Ad-ARKO females (X^2^ P=0.0001) that increases to 80% in G2 Ad-ARKO females (X^2^ P=0.0001) compared to only a 20% occurrence in WT controls (X^2^ P=0.5398), (n=5). For statistical analysis, observed vs. expected values were obtained from morphology analysis in WT controls (80% no hyperplasia and 20% hyperplasia), and were then used for the statistical analysis on G1 and G2 Ad-ARKO mice. All mice collected and analyzed at d80. Scale Bars 100 µm. Annotations; ZF, zona fasciculata; ZG, zona glomerulosa; X, X-zone.

In the adrenal, spindle cell hyperplasia is not uncommon in ageing mice and has been noted more commonly in females; however, the development of these cells in young mice is rare (26, 27). Consistent with increased adrenal weight and disrupted cortical markers, morphological analysis of G1 and G2 Ad-ARKO females reveals abnormal cell populations in the outer cortex at d80, which is classed as young adult (**Figures 4A–C**). The morphology of these cells is characteristic of spindle cells with an ovoid or elongated spindle shape classically named “type A cells” (**Figure 4C**) (27, 28). Analysis examining presence/absence of spindle cells reveals that at d80, 20% of control adrenal glands have spindle cell hyperplasia, with an increase to 60% in G1 Ad-ARKO females and a further increase to 80% in G2 Ad-ARKOs (**Figure 4D**).

### Cell Proliferation Is Perturbed in Ad-ARKO Females

**Figure 5:**
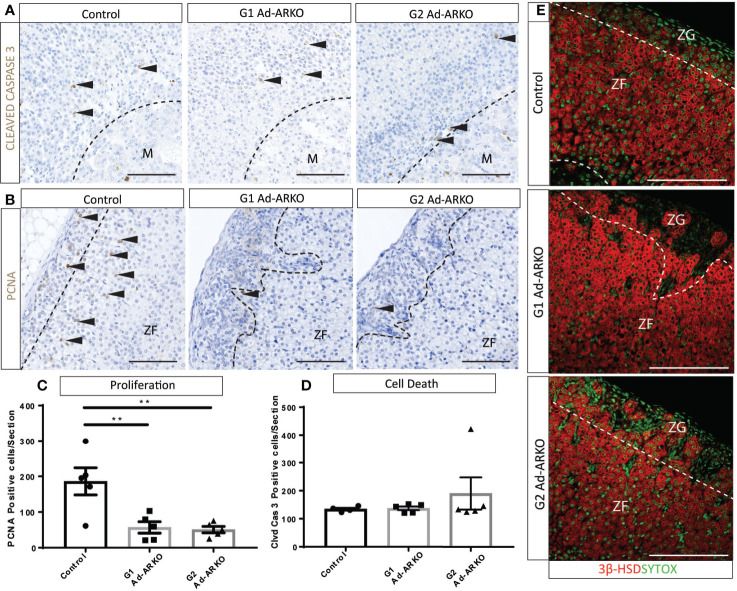
Fewer proliferating cells are observed on G1 and G2 Ad-ARKO females compared to WT controls. **(A, D)** Investigation of apoptosis revealed no changes in either G1 or G2 Ad-ARKO females when compared to WT controls. Confirmation through cell counts reveals no changes in cell death in G1or G2 Ad-ARKO females compared to WT controls. **(B, C)** Immunostaining of PCNA localization and cell counts reveal fewer proliferating cells in G1and G2 Ad-ARKO females compared to WT controls. **(E)** Immunostaining of 3βHSD localization reveals that sections of the ZG do not express 3βHSD in G1 or G2 Ad-ARKO females, compared to WT controls that are positive in both the ZG and ZF. (*) denotes insert location. N=5. All mice collected and analyzed at d80. Scale Bars 100 µm. Annotations; M, medulla; ZF, zona fasciculata; ZG, zona glomerulosa.

Normal development and cellular turnover of the adrenal cortex is tightly linked to the balance between proliferation, differentiation and cell death (29), due to the presence of spindle cells in the outer cortex, we would expect to see perturbed cellular turnover. It has been shown that spindle cells in the adrenal cortex proliferate from the sub-capsular region, and can extend into the ZF (30). Proliferation marker, PCNA (31) and apoptosis marker, CLEAVED CASPASE 3 (32), were analyzed to determine whether the spindle cells present in Ad-ARKO adrenals is a result of perturbed cellular turnover. Cell clearance from the adrenal cortex under normal conditions occurs at the cortex-medulla boundary (33), and immunostaining of cleaved caspase 3 shows no changes in location or frequency of apoptosis in any experimental cohort compared to controls (**Figures 5A, D****)**. Immunostaining and cell counts reveal significantly fewer proliferating cells in G1 and G2 Ad-ARKOs compared to controls (**Figures 5B, C****)**. Furthermore, in both G2 and G2 Ad-ARKOs, ZG cells no longer stain positive for steroid marker, 3β-HSD (**Figure 5E**).

## Discussion

Androgens have long been known to be essential regulators of male and female health and have been shown to influence adrenal remodeling (34, 35). Despite this, their role in adrenal cortex development and X-zone regulation has yet to be determined. For these reasons, we used a novel mouse model involving ablation of AR from the adrenal cortex through the use of a *Cyp11a1*-Cre (15). The data described herein demonstrate that AR is not required for the development of the adrenal cortex or X-zone regression observed during first pregnancy. Instead, AR signaling acts to suppress corticosterone levels in post-partum females. Furthermore, we demonstrate AR is required to prevent spindle cell hyperplasia in young adult females.

The role of the X-zone and the mechanisms controlling its maintenance or regression have to date, remained elusive. Control of X-zone regression has long been considered to be androgen dependent; this is due to early experiments showing treatment of WT male and female mice with testosterone-propionate, which results in X-zone regression. Conversely, gonadectomy has been shown to result in X-zone re-development (13, 36, 37). We recently further advanced this understanding by demonstrating that AR-signaling is essential for the normal pubertal X-zone regression in the male adrenal (14). Furthermore, we have demonstrated that AR localization in the female adrenal cortex during development and adulthood is similar to what is observed during adrenal development in the male. The X-zone in the female persists until first pregnancy when it then regresses, we thus hypothesized a similar role for androgen-AR signaling in this regression process, and therefore by ablating AR, we predicted that the X-zone in Ad-ARKO females would persist during and after pregnancy. Surprisingly, our results demonstrate that AR signaling is dispensable during X-zone regression in pregnancy and suggests that X-zone regression during pregnancy is controlled by an AR-independent mechanism. The rodent X-zone expresses 20-alpha HSD, an enzyme responsible for catabolizing progesterone (38). A small number of studies have assessed the impact of progesterone treatment on X-zone regulation and its contributions to progesterone regulation during pregnancy. Work carried out by Ungar *et al*., has demonstrated that although administration of testosterone does result in X-zone involution, treatment with progesterone was also able to induce regression but required a sustained dose over 6 days to do so (38). Given the sustained rise in circulating progesterone during pregnancy, we speculate that progesterone could play a significant role in X-zone regression in pregnant females; this requires further investigation.

An unexpected result of our study was the significant increase in corticosterone following pregnancy in G2 Ad-ARKO females. Previous studies have demonstrated that androgens are able to regulate corticosterone production (39), and development of postpartum depression has been associated with dysregulated HPA-axis and elevated cortisol levels (40). No disruption to corticosterone was observed in virgin female Ad-ARKOs. The chronic challenge placed on the HPA-axis during pregnancy and resulting elevated corticosterone levels observed in post-partum G2 Ad-ARKO females, suggests that ablation of AR does not result in perturbed HPA-axis function in basal conditions, however, may be required to suppress corticosterone levels in chronic stress conditions. Our results suggest a possible mechanistic link between suppressed androgen signaling and raised corticosteroid levels immediately post-partum that is worthy of further investigation.

It is well known that sex steroid receptors are essential for cell proliferation, differentiation and cell death. For these reasons, they have been an essential point of inquiry for the development of tumors in numerous tissues throughout the body (41–43). Our study highlights that spindle cells develop more frequently at an early age in both generations of female Ad-ARKOs, with a more pronounced phenotype observed in G2 females, when compared to controls. The mechanisms that lead to the development of spindle cells are of particular interest due to their association with the development of adrenocortical tumors (44, 45). A recent study has suggested that the low circulating androgens in females result in a preference for PKA-prone adrenal cortex differentiation and maintenance. Work carried out by Dumontet et al., demonstrated that androgens act as a negative regulator of the protein kinase A (PKA) pathway for normal cortex maintenance. The preference of this pathways in females means that cellular turnover is higher (full adrenal cortex cell replacement every 3 months (46)) compared to males (full adrenal cortex cell replacement 1 year (46)). Early development of spindle cells in Ad-ARKO females potentially suggests that the already low levels of circulating androgens combined with the loss of AR accelerates the early development of spindle cell hyperplasia. Given the presence of spindle cells, an unexpected result is the loss of proliferation in G1 and G2 Ad-ARKO mice. However, AR-signaling has been shown to be required for cell proliferation in numerous tissues throughout the body (47–49).

Of key interest, is the differences observed between male and female adrenal regulation by AR. This work contributes to mounting evidence that the mechanisms driving adrenal development, regulation and function are sex dependent. Spindle cell development in Ad-ARKO males did not present with spindle cell lesions until aged to 12 months (14) in comparison to Ad-ARKO females in which spindle cell lesions were observed by 3 months. However, analysis of AR localization during embryonic and postnatal development showed a similar spatiotemporal location in both WT males and females, suggesting an overlapping role during development.

Further work would be required to detail the cause/consequence relationship between AR-signaling and spindle cell development. Furthermore, we cannot at this stage comment on the benign or malignant properties of the spindle cells, however, these findings suggest that dysfunctional AR signaling promotes early spindle cell development, highlighting that the AR signaling pathway could act as an entry point to the wider understanding of adrenocortical tumor pathology. In conclusion, this study identifies essential and hitherto unknown roles for AR signaling in the female adrenal cortex regulation and function. These data demonstrate that AR-signaling is dispensable for adrenal development and pregnancy-driven X-zone regression, however, AR acts to suppress elevated corticosterone in post-partum females, support cell turnover and suppress early spindle cell formation. Together, these findings further our understanding of the role of androgens in the development and function of the adrenal gland.

## Data Availability Statement

The original contributions presented in the study are included in the article/supplementary materials; further inquiries can be directed to the corresponding author.

## Ethics Statement

The animal study was reviewed and approved by UK Home Office (License number 70/8804) held by LBS.

## Author Contributions

A-LG, LO, and LBS conceived and designed the study. A-LG, LO, SS, LM, and MC. carried out the experiments. A-LG, LO, IJM, RM, and LBS analyzed the results. AJ and HF provided novel resources. A-LG and LBS wrote the paper. All authors contributed to the article and approved the submitted version.

## Funding

This work was funded by a Medical Research Programme Grant Award (MR/N002970/1) (to LBS).

## Conflict of Interest

The authors declare that the research was conducted in the absence of any commercial or financial relationships that could be construed as a potential conflict of interest.
